# Effectiveness Factors and Conversion in a Biocatalytic Membrane Reactor

**DOI:** 10.1371/journal.pone.0153000

**Published:** 2016-04-22

**Authors:** Buntu Godongwana

**Affiliations:** Department of Chemical Engineering, Cape Peninsula University of Technology, Cape Town, South Africa; Brandeis University, UNITED STATES

## Abstract

Analytical expressions of the effectiveness factor of a biocatalytic membrane reactor, and its asymptote as the Thiele modulus becomes large, are presented. The evaluation of the effectiveness factor is based on the solution of the governing equations for solute transport in the two regions of the reactor, i.e. the lumen and the matrix (with the biofilm immobilized in the matrix). The lumen solution accounts for both axial diffusion and radial convective flow, while the matrix solution is based on Robin-type boundary conditions. The effectiveness factor is shown to be a function of the Thiele modulus, the partition coefficient, the Sherwood number, the Peclet number, and membrane thickness. Three regions of Thiele moduli are defined in the effectiveness factor graphs. These correspond with reaction rate limited, internal-diffusion limited, and external mass transfer limited solute transport. Radial convective flows were shown to only improve the effectiveness factor in the region of internal diffusion limitation. The assumption of first order kinetics is shown to be applicable only in the Thiele modulus regions of internal and external mass transfer limitation. An iteration scheme is also presented for estimating the effectiveness factor when the solute fractional conversion is known. The model is validated with experimental data from a membrane gradostat reactor immobilised with *Phanerochaete chrysosporium* for the production of lignin and manganese peroxidases. The developed model and experimental data allow for the determination of the Thiele modulus at which the effectiveness factor and fractional conversion are optimal.

## Introduction

Membrane bioreactors (MBR’s) offer a number of advantages over traditional bioreactors and their use for various bioconversions have been extensively reported [1–3]. The main challenge in the use of MBR’s remains the diffusional resistance of the membrane which adversely affects their performance [4,5]. The effectiveness factor (*η*), defined as the ratio of the observed rate of reaction to the hypothetical rate in the absence of mass transfer limitations [6], is generally used to evaluate the performance of a catalytic reactor. A thorough review of mathematical methods employed in evaluating exact solutions of this parameter was given by Aris [6]. This study presented effectiveness factors for single and multiple reactions taking place in various shapes of porous catalysts. Webster and co-workers [7,8] presented analytical models for a membrane bioreactor immobilized with whole cells, based on both Robin-type and Dirichlet-type boundary conditions. The former boundary type accounts for external mass transfer limitations, while the latter assumes the concentration at the membrane wall is known. Willaert *et al*. [9] obtained identical effectiveness factor expressions to Webster and Shuler [7] based on Dirichlet boundary conditions. In these studies, as well as in the majority of available exact solutions [10–12], axial diffusion and radial convective flows are neglected and the kinetics are generally considered linear. These assumptions are not always justified [13] and are imposed with the intention of attaining closed-form expression of the transport equation. The analytical solution of the mass balance equation is not always feasible, and a number of numerical schemes have been developed for this purpose [14–20]. Analytical models however are preferred for their simplicity.

**Fig 1:**
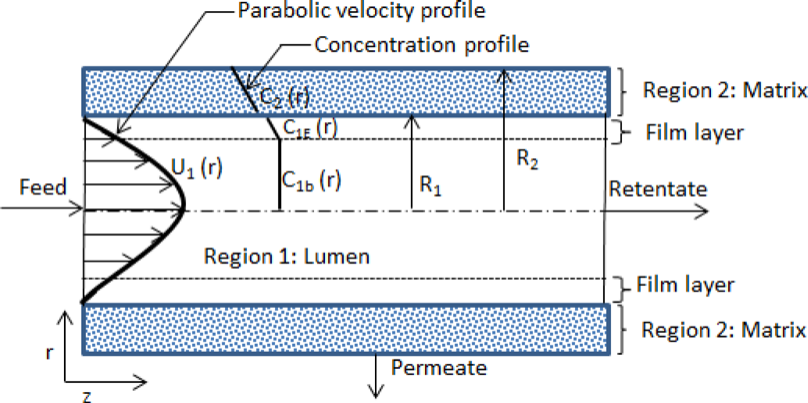
A cross-section of the membrane bioreactor illustrating the different regions of the MBR (i.e. lumen, film layer, and matrix). The velocity distribution and concentration distribution are also shown.

The current analysis is aimed at developing expressions of the effectiveness factor for an MBR immobilized with biofilm, based on the model developed by Godongwana *et al*. [13]. The asymptotic behaviour as the Thiele-modulus becomes large will be considered. The models are based on the MBR system shown in Fig 1, and the following conditions of operation are assumed: (1) the system is isothermal; (2) the flow regime within the membrane lumen is fully developed, laminar, and homogeneous; (3) the physical and transport parameters are constant; (4) in the membrane matrix the flow is only one dimensional (i.e. there are no axial components of the velocity in the membrane matrix).

## Mathematical Formulation

### Governing equations

The governing equations for solute transport in the lumen and matrix of the MBR are respectively:

$$u_{1}\frac{\partial c_{1}}{\partial z}+v_{1}\frac{\partial c_{1}}{\partial r}=D_{1}\left[\frac{1}{r}\frac{\partial }{\partial r}(r\frac{\partial c_{1}}{\partial r})+\frac{\partial^{2}c_{1}}{\partial z^{2}}\right]$$ (1)

$$\frac{D_{2}}{r}\frac{\partial }{\partial r}\left(r\frac{\partial c_{2}}{\partial r}\right)-v_{2}\frac{\partial c_{2}}{\partial r}=\frac{V_{M}c_{2}}{K_{m}+c_{2}}$$ (2)

The MBR is considered axisymmetric and the associated boundary conditions to Eqs (1) and (2) are:

$$B.C.1\;at\;z=0\;\forall r\;c_{1}=c_{0}$$ (3a)

$$B.C.2\;at\;r=0\;\forall z\;\frac{\partial c_{1}}{\partial r}=0$$ (3b)

$$B.C.3\;at\;r=R_{1}\;\forall z\;\frac{\partial c_{1}}{\partial z}=\frac{2D_{1}}{u_{1}R_{1}}\frac{\partial c_{1}}{\partial r}$$ (3c)

$$B.C.4\;at\;r=R_{1}\;\forall z\;k_{a}(c_{1b}-c_{1E})=-D_{2}\frac{\partial c_{2}}{\partial r}$$ (3d)

$$B.C.5\;at\;r=R_{2}\;\forall z\;\frac{\partial c_{2}}{\partial r}=0$$ (3e)

where *u* and *v* are the axial and radial velocity components, respectively; *c*_1_ and *c*_2_ are the local substrate concentrations in the lumen and fiber matrix, respectively; *c*_*1b*_ is the bulk lumen concentration; *c*_1E_ is the concentration on the internal surface of the membrane; *D*_1_ and *D*_2_ are the substrate diffusion coefficients in the lumen and matrix, respectively; *k*_*a*_ is the mass transfer coefficient; *K*_*m*_ is the saturation constant; and *V*_*M*_ is the maximum rate of reaction.

Boundary condition 1 (B.C.1) corresponds to a uniform inlet substrate concentration; B.C.2 corresponds to cylindrical symmetry at the centre of the membrane lumen; B.C.3 and B.C.4 corresponds to continuity of the substrate flux at the lumen-matrix interface. The partition coefficient for the transfer from outside the film layer to inside the film is assumed to be unity. Only the partition for the transfer from the film layer to the matrix is considered, as shown in Fig 1. B.C.5 implies there is no diffusion across the matrix-shell interface. In single-substrate limited biofilms, *V*_*M*_ in Eq (2) is given by [21]:

$$V_{M}=\frac{\mu_{max}X}{Y_{X/S}}$$ (4)

where *X* is the average biofilm density, *μ*_*max*_ is the maximum specific growth rate, and *Y*_*x/s*_ is the yield of biofilm per unit substrate.

### MBR lumen (Region 1)

In the lumen-side of the MBR, Eq (1) in dimensionless form becomes:

$$\varphi {Pe}_{u}U_{1}\frac{\partial C_{1}}{\partial Z}-\varphi^{2}\frac{\partial^{2}C_{1}}{\partial Z^{2}}=\frac{1}{R}\left(\frac{\partial C_{1}}{\partial R}+R\frac{\partial^{2}C_{1}}{\partial R^{2}}\right)-{Pe}_{v}V_{1}\frac{\partial C_{1}}{\partial R}$$ (5)

where:

$$U=\frac{u}{u_{0}};\;V=\frac{v}{v_{0}};\;C=\frac{c}{c_{0}};\;Z=\frac{z}{L};\;R=\frac{r}{R_{1}};\;\varphi =\frac{R_{1}}{L}$$

$${Pe}_{u}=\frac{u_{0}R_{1}}{D_{1}};\;{Pe}_{v}=\frac{v_{0}R_{1}}{D_{1}}$$ (6)

The solution of Eq (5) was given by Godongwana *et al*. [13] as an asymptotic expansion in terms of the membrane hydraulic permeability *κ*:

$$C_{1}(\theta ,x)=\sum_{m=1}^{\infty }\sum_{n=0}^{N}{B_{m}F}_{m}(\theta )T_{n}(x)\kappa^{n}$$ (7)

Where

$$\begin{array}{ccc} \theta =-\left(\frac{\varphi^{2}}{4{Pe}_{u}\kappa \beta }\right)\xi^{2}; & \xi =-\frac{2{Pe}_{u}\kappa \beta }{\varphi^{2}}\left[\frac{1}{\left(f-1\right)}+Z\right]; & and\;x=\lambda_{m}R \end{array}$$ (8)

and *F*_*m*_*(θ)* in Eq (7) is the Kummer function:

$$F_{m}(\theta )=M\left(-\frac{\lambda_{m}^{2}}{4{Pe}_{u}\kappa \beta },\frac{1}{2},\theta \right)$$ (9)

The zero-order and first-order approximations of *T*_*n*_*(x)* in Eq (7) are, respectively:

$$T_{0}(x)=J_{0}(x)$$ (10)

and

$$T_{1}(x)=\sigma_{1}\left[\frac{{\left(x\right)}^{2}J_{2}(x)}{3‼}+\sigma_{2}\frac{{\left(x\right)}^{3}J_{3}(x)}{5‼}+\sigma_{3}\frac{{\left(x\right)}^{4}J_{4}(x)}{7‼}\right]$$ (11)

where *λ*_*m*_ are the eigenvalues, *J*_*n*_ is the Bessel function of the first kind of order *n*.

$$\sigma_{1}=-\frac{3{Pe}_{v}\beta (\frac{u_{0}}{v_{0}})}{2\lambda_{m}^{2}},\;\sigma_{2}=-\frac{20}{3\lambda_{m}^{2}},\;and\;\sigma_{3}=\frac{35}{4\lambda_{m}^{2}}$$ (12)

The eigenvalues are obtained from B.C.3 in Eq (3c), and are roots of the equation [20]:

$$\frac{\lambda_{m}\varphi \xi }{\kappa \beta }M\left(-\frac{\lambda_{m}^{2}}{4{Pe}_{u}\kappa \beta }+1,\frac{3}{2},\theta \right)=4\frac{J_{1}(\lambda_{m})}{J_{0}(\lambda_{m})}M\left(\frac{-\lambda_{m}^{2}}{4{Pe}_{u}\kappa \beta },\frac{1}{2},\theta \right)$$ (13)

The coefficient *B*_*m*_ is obtained by imposing the inlet condition B.C.1 of Eq (3a):

$$B_{m}=\frac{2}{\lambda_{m}M(-\frac{\lambda_{m}^{2}}{4{Pe}_{u}\kappa \beta },\frac{1}{2},\theta_{0})}\left[\frac{J_{1}(\lambda_{m})}{J_{0}^{2}(\lambda_{m})+J_{1}^{2}(\lambda_{m})}\right]$$ (14)

### MBR Matrix (Region 2)

#### First-order Kinetics

The rate of solute consumption inside the membrane matrix is governed by Monod kinetics. Assuming the first-order limit, i.e. *K*_*m*_ >> *c*, Eq (2) for the matrix in dimensionless form becomes:

$$\frac{d^{2}C_{2}}{dR^{2}}+\left(\frac{1}{R}-{Pe}_{v}V_{2}\right)\frac{dC_{2}}{dR}-\phi^{2}C_{2}=0$$ (15)

where the first-order Thiele modulus *ϕ* is defined as:

$$\phi =\sqrt{\frac{V_{M}R_{1}^{2}}{K_{m}D_{2}}}$$ (16)

Eq (15) is amenable to an analytical solution by regular perturbation only when the hydraulic permeability is much smaller than unity *κ* << 1. For brevity only the zero-order approximation will be considered, the first order perturbation approximation is given in Appendix A following the procedure of Godongwana *et al*. [13]. Eq (15) then reduces to:

$$\frac{d^{2}C_{2}}{dR^{2}}+\frac{1}{R}\frac{dC_{2}}{dR}-\phi^{2}C_{2}=0$$ (17)

Eq (17) is evaluated subject to B.C.4, which in dimensionless form becomes:

Sh(Cb−C2γ)=−dC2dR|R=1 (18)

Where *γ* is the partition coefficient and *Sh* is the Sherwood number. A good estimate of *Sh* for hollow fiber membranes is given by Wickramasinghe *et al*. [22]:

$$Sh=1.11{Re}^{0.47}{Sc}^{0.33}$$ (19)

where *Sc* = *μ*/*ρD*_*AB*_ is the Schmidt number and *Re* = *ρvR*_1_/*μ* is the Reynolds number. The dimensionless bulk lumen concentration is defined as:

$$C_{b}=2\int_{0}^{1}C_{1}(\theta ,x)RdR=2\sum_{m=1}^{\infty }\frac{B_{m}}{\lambda_{m}}∙M(-\frac{\lambda_{m}^{2}}{4{Pe}_{u}\kappa \beta },\frac{1}{2},\theta_{1})∙J_{1}(\lambda_{m})$$ (20)

Eq (17) is the modified Bessel’s equation and has a solution of the form [23]:

$$C_{2}=B_{1}I_{0}(\phi R)+B_{2}K_{0}(\phi R)$$ (21)

where *I*_*0*_ and *K*_*0*_ are the modified Bessel functions of the first kind and second kind, respectively. The constants *B*_*1*_ and *B*_*2*_ are obtained with the use of B.C.4 and B.C.5 as:

$$B_{1}=\frac{K_{1}(\phi R_{2})∙\gamma C_{b}}{[K_{0}(\phi )∙I_{1}(\phi R_{2})+I_{0}(\phi )∙K_{1}(\phi R_{2})]+\psi }$$ (22)

and

$$B_{2}=\frac{I_{1}(\phi R_{2})∙\gamma C_{b}}{[K_{0}(\phi )∙I_{1}(\phi R_{2})+I_{0}(\phi )∙K_{1}(\phi R_{2})]+\psi }$$ (23)

where

$$\psi =\frac{\gamma \phi }{Sh}[K_{1}(\phi )∙I_{1}(\phi R_{2})+I_{1}(\phi )∙K_{1}(\phi R_{2})]$$ (24)

The effectiveness factor is defined as:

$$\eta =\frac{-2\pi R_{1}LD_{2}{\frac{\partial c_{2}}{\partial r}|}_{r=R_{1}}}{\pi L(R_{2}^{2}-R_{1}^{2})\frac{V_{M}c_{b}}{K_{m}+c_{b}}}$$ (25)

In dimensionless form:

$$\eta =\frac{-2(\delta +1)}{\phi_{0}^{2}(R_{2}^{2}-1)}{\frac{\partial C_{2}}{\partial R}|}_{R=1}$$ (26)

where $\delta =\frac{K_{m}^{*}}{C_{b}}$ and *ϕ*_0_ is the zero-order Thiele modulus defined as:

$$\phi_{0}=\sqrt{\frac{V_{M}R_{1}^{2}}{c_{0}D_{2}}}$$ (27)

Assuming first-order kinetics (*δ* >> 1) Eq (26) reduces to:

$$\eta_{1}=\frac{-2}{\phi^{2}(R_{2}^{2}-1)C_{b}}{\frac{\partial C_{2}}{\partial R}|}_{R=1}$$ (28)

Substituting Eqs (20–24) into Eq (28) gives:

$$\eta_{1}=\frac{2\gamma [{K_{1}(\phi )∙I_{1}(\phi R_{2})-I}_{1}(\phi )∙K_{1}(\phi R_{2})]}{\phi (R_{2}^{2}-1)\{[K_{0}(\phi )∙I_{1}(\phi R_{2})+I_{0}(\phi )∙K_{1}(\phi R_{2})]+\psi \}}$$ (29)

The reciprocal of the effectiveness factor is generally considered a mass transfer resistance [6,8,24]. Thus, the reciprocal of Eq (29) is the sum of the internal resistance and the external resistance (*ψ*) to mass transfer. This is explicit in the asymptotic form of Eq (29) given in the Appendix B:

$$\begin{array}{cc} \frac{1}{\eta_{1}}=\frac{\phi (R_{2}^{2}-1)}{2}\left\{\frac{\phi }{Sh}+\frac{1}{\gamma }coth[\phi (R_{2}-1)]\right\} & ,as\;\phi \to \infty \end{array}$$ (30)

The first and second terms inside the curly brackets in Eq (30) represent the external resistance and internal resistance to mass transfer, respectively. The series-of-resistances nature of Eqs (29) and (30) is a result of using the Robin-type boundary condition, B.C.4, in the evaluation of Eq (17). In both equations the parameters with the greatest influence on the effectiveness factor are: the Thiele modulus, partition coefficient, Sherwood number, and membrane thickness. The influence of the Peclet (*Pe*_*u*_) number on the effectiveness factor is presented in Appendix A. By definition *η* = 1 when the Thiele modulus *ϕ* becomes zero since this value of the Thiele modulus corresponds with a reaction rate-controlled transfer with no mass transfer limitations.

#### Zero-order Kinetics

Assuming the zero-order limit, i.e. *K*_*m*_ << *c*, the dimensionless form of Eq (2) becomes:

$$\frac{d^{2}C_{2}}{dR^{2}}+\frac{1}{R}\frac{dC_{2}}{dR}-\phi_{0}^{2}=0$$ (31)

Eq (31), subject to B.C. 4 and B.C.5, has a solution of the form:

$$C_{2}=\frac{\phi_{0}^{2}}{4}\left\{(R^{2}-1)-2[R_{2}^{2}lnR+\frac{\gamma }{Sh}(R_{2}^{2}-1)]\right\}+\gamma C_{b}$$ (32)

The dimensionless zero-order effectiveness factor from Eq (26) is:

$$\eta_{0}=-\frac{2}{\phi_{0}^{2}(R_{2}^{2}-1)}{\frac{\partial C_{2}}{\partial R}|}_{R=1}=1$$ (33)

#### Non-linear Kinetics

The effectiveness factor allows for the determination of the overall reaction rate in terms of the Thiele modulus. However, when the reaction kinetics are not linear as was assumed in the above analysis Eq (26) is not amenable to an analytical solution. A practical measure of evaluating the effectiveness factor is attained by making the following approximation:

$${\frac{dC_{2}}{dR}|}_{R=1}=\begin{array}{cc} \frac{{C_{2}|}_{{R=R}_{2}}-{C_{2}|}_{R=1}}{\left(R_{2}-1\right)}, & (R_{2}-1)\ll 1 \end{array}$$ (34)

Substituting Eq (34) into Eq (26) gives:

$$\Upsilon =\begin{array}{cc} 1-\left[{C_{2}|}_{R=1}-\frac{\eta \phi_{0}^{2}(R_{2}^{2}-1)(R_{2}-1)}{2(\delta +1)}\right], & (R_{2}-1)\ll 1 \end{array}$$ (35)

where *Υ* is the fractional conversion. Eq (35) allows for empirical determination of the effectiveness factor when the fractional conversion is known, from the following procedure: (i) guess the wall concentration (${C_{2}|}_{{R=R}_{1}}$) and obtain the concentration gradient from Eq (34), (ii) substitute the concentration gradient ${\frac{dC_{2}}{dR}|}_{R=1}$ into Eq (26) to obtain the effectiveness factor, (iii) substitute the effectiveness factor *η* into Eq (35) and compare the experimental conversion to the attained value, and (iv) repeat the procedure until the experimental conversion is equal to the value obtained from the iteration.

## Results

**Fig 2:**
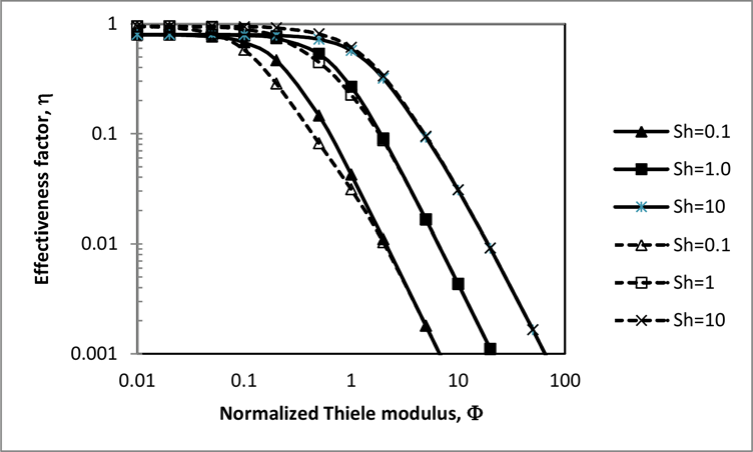
Effectiveness factors (—) from Eq (29) and asymptotes (—) from Eq (30) vs Thiele modulus at different Sherwood numbers.

Fig 2 is a plot of effectiveness factors and corresponding asymptotes, from Eqs (29) and (30) respectively, as functions of the normalized Thiele modulus *Φ* for different values of the Sherwood number. The normalized modulus is defined as:

$$\Phi =\frac{\phi }{2}(R_{2}^{2}-1)$$ (36)

Eq (30) provides a simple mathematical approximation to Eq (29) and for *Φ* > 1 gives exact values for the effectiveness factor, as shown in Fig 2. Three regions of Thiele moduli may be defined from Fig 2, as characterised by the effectiveness factor. In the first region (*Φ* < 0.01) the effectiveness factor is unity, and the rate of solute transport in the MBR is controlled by the rate of reaction. When the MBR is operated in this region the diffusional resistance offered by the membrane is negligible. In the second region (0.01 < *Φ* < 0.1) the rate of solute transport is limited only by internal diffusion through the membrane, and hence the effectiveness factor is not a function of the Sherwood number. In the third region (*Φ* > 0.1) external mass transfer limitations control the rate of solute transport through the MBR, and the effectiveness factor is greatly influenced by the Sherwood number. This result is consistent with the Robin-type boundary condition.

**Fig 3:**
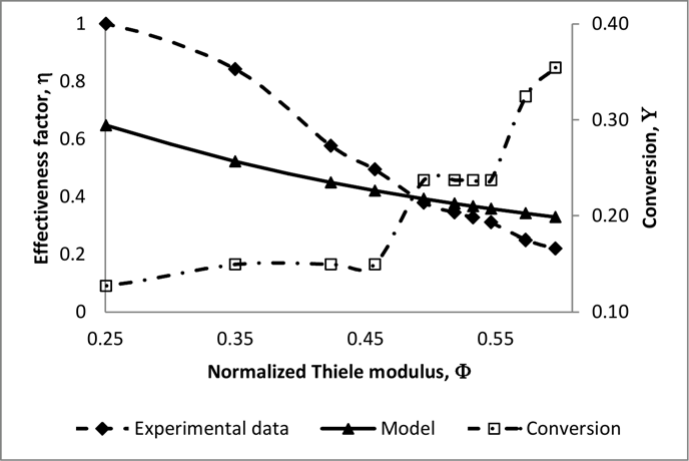
Effectiveness factor and glucose conversion vs normalized Thiele modulus (Sh = 0.83).

Fig 2 may suggest operating the MBR at low values of the Thiele modulus for high effectiveness factors, however substrate conversion at these low values is minimal as can be seen in Fig 3. This figure presents experimental values of conversion and the effectiveness factor for an MBR used for the production of Lignin and Manganese Peroxidases from *Phanerochaete chrysosporium*. The operating parameters of the MBR and kinetic constants of the biofilm are listed in Table 1. From Fig 3 an operating Thiele modulus may be found at which both substrate conversion and the effectiveness factor are optimal. This point corresponds with low effectiveness factors when the objective is to maximise solute conversion [19].

**Table 1:** Parameter values used to determine the effectiveness factor in Fig 3 [25].

Model parameter	Symbol	Unit	Basic measured value
Membrane inner radius	*R*_*1*_	m	6.98 x 10^−4^
Membrane outer radius	*R*_*2*_	m	9.63 x 10^−4^
Effective membrane length	*L*	m	0.230
Lumen-side entrance velocity	*u*_*0*_	ms^-1^	3.04 x 10^−4^
Permeation velocity	*v*_*0*_	m s^-1^	8.82 x 10^−6^
Glucose diffusivity	*D*_*AB*_	m^2^ s	1.59 x 10^−9^
Glucose inlet concentration	*c*_*0*_	g dm^-3^	10.00
Maximum specific growth rate	*μ*_*max*_	h^-1^	0.035
Saturation constant	*K*_*m*_	g dm^-3^	9.350
Yield of biofilm per substrate	*Y*_*x/s*_	g/g	0.202

The experimental effectiveness factor in Fig 3 is obtained from Eq (35) and is plotted against the first-order model of Eq (29). The two plots exhibit the same trend, with the model underestimating the effectiveness factor at values of Φ < 0.5. This is because at low values of the Thiele modulus solute transport is reaction rate controlled and the first-order kinetics premise assumes a lower rate of reaction than the maximum. At higher values of the Thiele modulus solute transport is limited by internal and external diffusion, and the first-order model approximately matches the experimental effectiveness factor.

**Fig 4:**
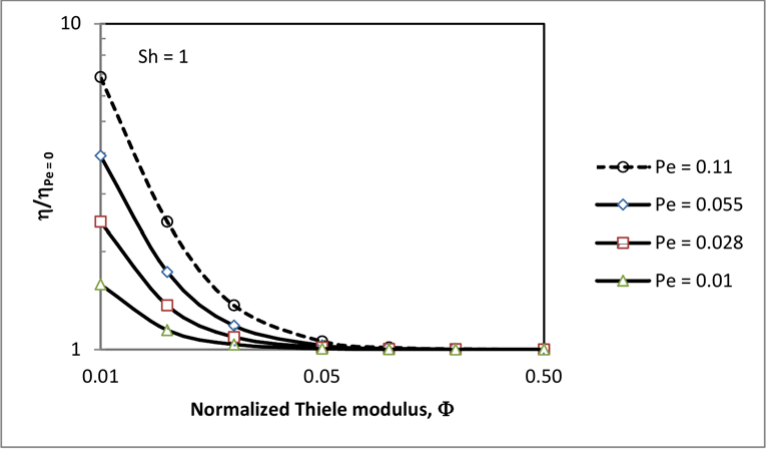
Relative increase in effectiveness factor vs normalized Thiele modulus at different Peclet numbers.

In the region of internal diffusional limitation (0.01 < *Φ* < 0.1) radial convective flows can significantly improve the effectiveness factor, as illustrated in Fig 4. In this figure the relative increases in the effectiveness factor (ηηPe=0) are plotted against normalised Thiele moduli for different values of the radial Peclet number. The effectiveness factors in Fig 4 are obtained from Eq (A8) in Appendix A. The increase in *η* with *Pe*_*v*_ is only restricted to the region of internal diffusional limitation. The maximum relative increase in the effectiveness factor is obtained in the transitional region from kinetic to internal-diffusional control (*Φ* ≈ 0.01), and minimal in the boundary region between internal-diffusional control and external mass transfer limitation. Increasing *Pe*_*v*_ outside this region may drastically reduce the contact time between the substrate and the biocatalyst, and hence lead to reduced substrate conversions as was shown by Calabro *et al*. [17]. In this region (*Φ* > 0.1), as previously discussed the effectiveness factor can be improved by increased Sherwood numbers.

## Conclusion

Mathematical models were developed for solute concentration profiles and effectiveness factors in an MBR, assuming the zero-order and first-order limits of the Michaelis-Menten (or Monod) equation. The first-order kinetic model was shown to be applicable only when the MBR is operated at high Thiele moduli. Experimental results show that the effectiveness factor decreases with increasing Thiele modulus, while the fractional conversion increases with an increase in this parameter. The developed model allows for the determination of the operating point at which both the conversion and effectiveness factor are optimal. It was also shown that the radial Peclet number can significantly improve the performance of an MBR operating under internal diffusional limitations.

## Appendix A

### First-order perturbation approximation of the effectiveness factor

The solution of Eq (15) may be approximated by an asymptotic expansion:

$$C_{2}=C_{2}^{\left(0\right)}+\kappa C_{2}^{\left(1\right)}+\kappa^{2}C_{2}^{\left(2\right)}+\ldots \kappa^{n}C_{n}^{\left(n\right)}$$ (A1)

The zero-order approximation $C_{2}^{\left(0\right)}$ was given in Section 2.3 as:

$$C_{2}^{\left(0\right)}=B_{1}I_{0}(\phi R)+B_{2}K_{0}(\phi R)$$ (A2)

The first-order approximation $C_{2}^{\left(1\right)}$ is a solution of the equation:

$$\frac{d^{2}C_{2}^{\left(1\right)}}{dR^{2}}+\frac{1}{R}\frac{dC_{2}^{\left(1\right)}}{dR}+\phi^{2}C_{2}^{\left(1\right)}=2{Pe}_{u}\beta \left[R(1-\frac{R^{2}}{2})\right]\frac{dC_{2}^{\left(0\right)}}{dR}$$ (A3)

The modified Bessel function *K*_*v*_(*x*) tends to zero as |*x*| → ∞ for all values of *v*. The contribution of this function in Eq (A3) is therefore only significant as x → 0. In this region the limiting form of *K*_*v*_(*x*) is [23]:

$$\begin{array}{cc} K_{v}(x)\sim \frac{1}{2}\Gamma (v){\left(\frac{1}{2}x\right)}^{-v} & \left(v>0\right) \end{array}$$ (A4)

where *Γ*(*n*) is the Gamma function. The solution of Eq (A3) follows the same procedure as Godongwana *et al*. [13], and is of the form:

$$C_{2}^{\left(1\right)}=\frac{{Pe}_{u}\beta \kappa }{\phi^{2}}\left\{\frac{3\sqrt{\pi }B_{1}}{2}[\frac{{\left(\phi R\right)}^{2}I_{2}(\phi R)}{2^{2}\Gamma (2\frac{1}{2})}+\alpha_{1}\frac{{\left(\phi R\right)}^{3}I_{3}(\phi R)}{2^{3}\Gamma (3\frac{1}{2})}+\alpha_{2}\frac{{\left(\phi R\right)}^{4}I_{4}(\phi R)}{2^{4}\Gamma (4\frac{1}{2})}]-\frac{B_{2}}{\phi^{2}}[{{\left(\phi R\right)}^{2}-2\phi }^{2}+4]\right\}$$ (A5)

where

$$\alpha_{1}=-\frac{20}{3\phi^{2}},\;and\;\alpha_{2}=-\frac{35}{4\phi^{2}}$$ (A6)

The effectiveness factor is obtained by substituting the derivatives of Eqs (A2) and (A5) into Eq (28), making use of the following property of Bessel functions [23]:

$${\left(\frac{1}{z}\frac{d}{dz}\right)}^{k}\{z^{v}I_{v}(z)\}=z^{v-k}I_{v-k}(z)$$ (A7)

This gives:

$$\eta =\frac{2\gamma [{K_{1}(\phi )∙I_{1}(\phi R_{2})-I}_{1}(\phi )∙K_{1}(\phi R_{2})-\xi ]}{\phi (R_{2}^{2}-1)\{[K_{0}(\phi )∙I_{1}(\phi R_{2})+I_{0}(\phi )∙K_{1}(\phi R_{2})]+\psi \}}$$ (A8)

where

$$\xi ={Pe}_{u}\beta \kappa \left\{\frac{3\sqrt{\pi }∙K_{1}(\phi R_{2})}{8}[\frac{I_{1}(\phi )}{\Gamma (2\frac{1}{2})}+\alpha_{1}\frac{\phi I_{2}(\phi )}{2\Gamma (3\frac{1}{2})}+\alpha_{2}\frac{\phi^{2}I_{3}(\phi )}{4\Gamma (4\frac{1}{2})}]-\frac{2I_{1}(\phi R_{2})}{\phi^{3}}\right\}$$ (A9)

## Appendix B

### Asymptotic solution of the Effectiveness factor (*ϕ* → *∞*)

Eq (17) may be written as:

$$\frac{\epsilon^{2}}{R}\frac{d}{dR}\left(R\frac{dC_{2}}{dR}\right)-C_{2}=0$$ (B1)

where:

$$\epsilon =\frac{1}{\phi }$$ (B2)

The solution of Eq (17) may be approximated by an asymptotic expansion when *ϵ* ≪ 1 as:

$$C_{2}=b_{0}+\epsilon b_{1}+\epsilon^{2}b_{2}+\ldots$$ (B3)

In order to keep the second-order derivative in the solution of the coefficient *b*_0_ in Eq (B3), the following variable is defined:

$$\omega =\frac{1-R}{\epsilon }$$ (B4)

Eq (B1) then becomes:

$$\frac{d^{2}C_{2}}{d\omega^{2}}-\frac{\epsilon }{\left(1-\epsilon \omega \right)}\frac{dC_{2}}{d\omega }-C_{2}=0$$ (B5)

The leading order term sub-problem is:

$$\frac{d^{2}b_{0}}{d\omega^{2}}-b_{0}=0$$ (B6)

The corresponding boundary conditions are B.C.4 and B.C.5 of Eq (3):

$${\frac{db_{0}}{d\omega }|}_{\omega =0}=\epsilon Sh\left[C_{b}-\frac{b_{0}(0)}{\gamma }\right]$$ (B7a)

and

$${\frac{db_{0}}{d\omega }|}_{\omega =\left(1-R_{2}\right)/\epsilon }=0$$ (B7b)

The solution of Eq (B6), subject to the boundary conditions of Eq (B7) is:

$$b_{0}=\Lambda_{1}e^{\omega }+{\Lambda_{2}e}^{-\omega }$$ (B8)

where

$$\Lambda_{1}=\frac{1}{\phi (1+\frac{Sh}{\phi \gamma })}\left\{\frac{(1-\frac{Sh}{\phi \gamma })ShC_{b}e^{-[\phi (R_{2}-1)]}}{sinh[\phi (R_{2}-1)]+\frac{Sh}{\phi \gamma }cosh[\phi (R_{2}-1)]}+ShC_{b}\right\}$$ (B9)

And

$$\Lambda_{2}=\frac{1}{\phi }\left\{\frac{ShC_{b}e^{-[\phi (R_{2}-1)]}}{sinh[\phi (R_{2}-1)]+\frac{Sh}{\phi \gamma }cosh[\phi (R_{2}-1)]}\right\}$$ (B10)

The effectiveness factor is obtained by taking the derivative of Eq (B8) and substituting into Eq (28) to obtain:

$$\frac{1}{\eta_{1}}=\frac{\phi (R_{2}^{2}-1)}{2}\left\{\frac{\phi }{Sh}+\frac{1}{\gamma }coth[\phi (R_{2}-1)]\right\}$$ (B11)

## Abbreviations

B_m_: constants of integration of Bessel’s equation, *m* = 1, 2
c: substrate concentration (g dm^-3^)
c_b_: bulk lumen concentration (g dm^-3^)
*c*_0_: substrate feed concentration (g dm^-3^)
*C* = *c*/*c*_0_: dimensionless substrate concentration
D_AB_: substrate diffusivity (m^2^ s^-1^)
*f* = *u*_1_/*u*_0_: fraction retentate
*J*_*n*_(*λ*): Bessel function of order *n* of the first kind
k_a_: mass transfer coefficient (m s^-1^)
k_m_: membrane hydraulic permeability (m Pa^-1^s^-1^)
K_m_: saturation (or Monod constant) (g dm^-3^)
$K_{m}^{*}=K_{m}/c_{0}$: dimensionless Monod constant
L: membrane effective length (m)
*M*(*a*,*b*,*θ*): Kummer function of the first kind
*Pe*_*u*_ = *u*_0_*R*_*1*_/*D*_*AB*_: axial Peclet number
*Pe*_*v*_ = *v*_0_*R*_*1*_/*D*_*AB*_: radial Peclet number
r: radial spatial coordinate (m)
*R* = *r*/*R*_*1*_: dimensionless radial spatial coordinate
R_1_: membrane lumen radius (m)
*Re* = *ρu*_0_*R*_*1*_/*μ*: Reynolds number
Sc = μ/ρD_AB_: Schmidt number
Sh = k_a_R_1_/D_AB_: Sherwood number
u: axial velocity (m s^-1^)
*u*_0_: feed axial velocity (m s^-1^)
*U* = *u*/*u*_0_: dimensionless axial velocity
v: radial velocity (m s^-1^)
*v*_0_ = *k*_*m*_(*p*_0_—*p*_*S*_): permeation velocity (m s^-1^)
*V* = *v*/*v*_0_: dimensionless radial velocity
V_M_: maximum rate of reaction (g dm^-3^ s^-1^)
X: average biofilm density (g dm^-3^)
Y_x/s_: yield of biofim per unit substrate
z: axial spatial coordinate (m)
*Z* = *z*/*L*: dimensionless axial spatial coordinate
β: dimensionless transmembrane pressure
γ: membrane partition coefficient
*δ* = *K*_*m*_/*c*_0_*C*_*b*_: modified dimensionless Monod constant
$\epsilon =\frac{1}{\phi }$: substitution variable
η: effectiveness factor for general kinetics
*η*_0_: effectiveness factor for zero-order kinetics
*η*_1_: effectiveness factor for first-order kinetics
θ: substitution variable
$\kappa =\mu k_{m}L/R_{1}^{2}$: dimensionless membrane hydraulic permeability
λ_m_: eigen values, *m* = 1, 2, …
μ: solution dynamic viscosity (Pa s)
μ_max_: maximum specific growth rate (s^-1^)
ρ: solution density (kg m^-3^)
*φ* = *R*_1_/*L*: aspect ratio
ϕ: Thiele modulus
ψ: external resistance to mass transfer
Υ: fractional conversion
